# Sp5 induces the expression of Nanog to maintain mouse embryonic stem cell self-renewal

**DOI:** 10.1371/journal.pone.0185714

**Published:** 2017-09-29

**Authors:** Ling Tang, Manman Wang, Dahai Liu, Mengting Gong, Qi-Long Ying, Shoudong Ye

**Affiliations:** 1 Center for Stem Cell and Translational Medicine, School of Life Science, Anhui University, Hefei, PR China; 2 Eli and Edythe Broad Center for Regenerative Medicine and Stem Cell Research at USC, Department of Stem Cell Biology and Regenerative Medicine, Keck School of Medicine, University of Southern California, Los Angeles, California, United States of America; University of Texas at Austin Dell Medical School, UNITED STATES

## Abstract

Activation of signal transducer and activator of transcription 3 (STAT3) by leukemia inhibitory factor (LIF) maintains mouse embryonic stem cell (mESC) self-renewal. Our previous study showed that trans-acting transcription factor 5 (Sp5), an LIF/STAT3 downstream target, supports mESC self-renewal. However, the mechanism by which Sp5 exerts these effects remains elusive. Here, we found that Nanog is a direct target of Sp5 and mediates the self-renewal-promoting effect of Sp5 in mESCs. Overexpression of *Sp5* induced *Nanog* expression, while knockdown or knockout of *Sp5* decreased the *Nanog* level. Moreover, chromatin immunoprecipitation (ChIP) assays showed that Sp5 directly bound to the Nanog promoter. Functional studies revealed that knockdown of *Nanog* eliminated the mESC self-renewal-promoting ability of Sp5. Finally, we demonstrated that the self-renewal-promoting function of Sp5 was largely dependent on its zinc finger domains. Taken together, our study provides unrecognized functions of Sp5 in mESCs and will expand our current understanding of the regulation of mESC pluripotency.

## Introduction

Embryonic stem cells (ESCs) are derived from the inner cell mass (ICM) of the pre-implantation blastocyst [1]. ESCs were first established from mice [2, 3] and then from rats [4, 5]. ESCs can be maintained indefinitely as self-renewing populations while retaining the capacity to generate any cell type in the body; they not only have become a vital model system and powerful tool for understanding biological development and human diseases but also hold great promise for tissue repair and regeneration. Therefore, it is critical to understand more about how the ESC state is established and maintained. Extrinsic signals and intrinsic transcriptional circuitries govern ESC fate decisions. Notably, exogenous provision of leukemia inhibitory factor (LIF) maintains mESC self-renewal by activating signal transducer and activator of transcription 3 (STAT3) [6–8]. Extensive studies have identified many Stat3 downstream targets, such as Klf4, Gbx2, *Pim1*, *Pim3*, *Pramel7*, *c-Myc*, *Tfcp2l1* and *Sp5* [9–17]. Each can reproduce the self-renewal-promoting effect of LIF in mESCs when overexpressed. However, the specific mechanism by which they function in mESCs remains unclear.

Our previous report showed that *Sp5* is a downstream target of STAT3 and overexpression of *Sp5* is sufficient to maintain the undifferentiated state of mESCs in the absence of LIF [15]. Sp5, a member of the Sp1 family, is characterized by the presence of three typical zinc finger domains belonging to the specificity protein/Krϋppel-like factor (Sp/Klf) superfamily [18]. It binds to GC/GT-rich regions in the promoter of many genes to mediate the activation and/or repression of transcription [19, 20]. Sp5 plays key roles in many critical biological processes, including stem cell maintenance, cell proliferation, apoptosis, differentiation, and development, and represents a potential target for cancer therapy [21–25]. However, it is still unknown how Sp5 supports mESC self-renewal. Here, we showed that the effect of Sp5 on mESC self-renewal could be interrupted by *Nanog* knockdown. Furthermore, we demonstrate that Sp5 binds to the Nanog promoter to regulate its expression, indicating that Sp5 is an upstream activator of Nanog. In addition, we found that C-terminal zinc finger domains were indispensable for the full activity of Sp5 in mESCs. Collectively, our results provide a mechanism in which Sp5 acts as a mediator linking the LIF/STAT3 signaling pathway with Nanog to control mESC self-renewal and pluripotency.

## Materials and methods

### Cell culture

46C mESCs [26], which were provided by Qi-Long Ying (University of Southern California, USA), were cultured on 0.1% gelatin-coated dishes at 37°C in 5% CO_2_. The basal media for routine maintenance was Dulbecco's Modified Eagle Medium (DMEM, TransGen Biotech, China) supplemented with 10% Fetal Bovine Serum (FBS, ExCell Bio, Australia), 1× MEM non-essential amino acids (Invitrogen, USA), 2 mM GlutaMax (Invitrogen, USA), 1× sodium pyruvate (Invitrogen, USA), 0.1 mM β-mercaptoethanol (Invitrogen, USA), 1× penicillin/streptomycin (Invitrogen, USA), and 100 units/ml LIF (Millipore, USA). 293T cells were cultured in the same 10% FBS-DMEM except in the absence of LIF.

### Overexpression and knockdown plasmid construction

The coding region of *Sp5* was cloned from mESC cDNA with KOD Kit (Novagen, Japan) and inserted into the PiggyBac vector. Overlapping PCR was used to generate *Sp5* mutants. For RNA interference in mESCs, shRNA constructs were designed to target 21 base-pair gene-specific regions of *Sp5* and were then cloned into plko.1-TRC (AgeI and EcoRI sites). The targeted sequences are as follows:

*Sp5* sh#1: GGATTCAAAGGATTTGCTTTC;

*Sp5* sh#2: GGACTTTGCACAGTACCAGAG;

*Sp5* sh#3: GACTTTGCACAGTACCAGAGC;

*Nanog* sh#1: GGAGTATCCCAGCATCCATTG; and

*Nanog* sh#2: GACTAGCAATGGTCTGATTCA.

### Cell transfection and virus production

For gene overexpression, mESCs were transfected with 2 μg PiggyBac vectors inserted with genes plus 2 μg transposase vector using LTX (Invitrogen, USA) according to the manufacturer’s instructions. For the knockdown experiment, Plko.1-TRC-based lentiviral vectors and packaging plasmids (pCMV-VSVG and psPAX2) were transfected into 293T cells using LTX (Invitrogen, USA). Supernatant was collected after 48 h and passed through 0.45 μm filters (Millipore, USA). mESCs were cultured in the viral supernatant in the presence of 8 μg/ml polybrene (Sigma, USA) for 24 h. Selection began the next day by adding 2 μg/ml puromycin or 8 μg/ml blasticidin for 1 week.

### Generation of *Sp5*-knockout mESCs

The gRNA sequences designed according to the exon region of Sp5 were ligated into the pX330 vector (Addgene) and transduced into cells using LTX (Invitrogen, USA). Selection was continued for 1 week by adding 2 μg/ml puromycin. The targeted sequences are as follows:

Sp5 gRNA1F: CACCGCTGCAGTGAGTCGTTCCGG;

Sp5 gRNA1R: AAACCCGGAACGACTCACTGCAGC;

Sp5 gRNA2F: CACCGCTCCGGAACGACTCACTGC;

Sp5 gRNA2R: AAACGCAGTGAGTCGTTCCGGAGC;

Sp5 gRNA3F: CACCGGGCCGCTGTGGCCGTCCTC; and

Sp5 gRNA3R: AA ACGAGGACGGCCACAGCGGCCC.

### Embryoid body (EB) formation

For the EB formation assay, 1×10^7^ mESCs were grown using low-attachment dishes in standard mESC basal media in the absence of LIF or inhibitors. The aggregates were allowed to grow for 6 days and collected for qRT-PCR analysis.

### qRT-PCR

Total RNA was extracted using the TRIzol Up Plus RNA Kit (TransGen Biotech, China). cDNA was synthesized from 1 μg total RNA using the TransScript All-in-One First-Strand cDNA Synthesis SuperMix for qPCR (One-Step gDNA Removal, TransGen Biotech, China) according to the manufacturer’s instructions. qRT-PCR was carried out with Top Green qPCR SuperMix (TransGen Biotech, China) in a PikoReal Real-Time PCR machine (Thermo Scientific, USA). Target gene expression was normalized to β-actin expression. The primers used are listed in S1 Table.

### Western blot analysis

Cells were lysed in ice-cold RIPA cell buffer (Sigma, USA) supplemented with protease inhibitors (TransGen Biotech, China). Proteins were separated on a 10% PAGE gel (made in-house) and electrotransferred onto a PVDF membrane. Probing was performed with specific primary antibodies and HRP-conjugated secondary antibodies. The primary antibodies used were HA (3724S, Cell Signaling Technology, 1:1000), Flag (M2, Sigma, 1:1000) and α-Tubulin (SC-8035, Santa Cruz Biotechnology, 1:1000).

### Alkaline phosphatase activity assay

The alkaline phosphatase activity of mESCs cultured on gelatin-coated plates was detected using the Alkaline Phosphatase Kit (Sigma, USA).

### Immunofluorescence staining

Cells were fixed in 4% paraformaldehyde for 30 min and incubated at 37°C in blocking buffer (PBS containing 5% BSA and 0.2% Triton X-100). Cells were incubated in the presence of primary antibodies at 4°C overnight and then washed three times with PBS. Cells were then incubated with the Alexa Fluor 488 (Invitrogen, 1:1000) secondary antibody for 1 h at 37°C. Nuclei were stained with Hoechst (Invitrogen, 1:5000). The primary antibodies and dilutions used were Oct4 (sc-5279, Santa Cruz Biotechnology, 1:200), SSEA1 (sc-21702, Santa Cruz Biotechnology, 1:100) and Nanog (ab808692, Abcam, 1:100).

### Luciferase reporter assay

Different fragments of the Nanog promoter were inserted into the pGL3-basic plasmid (Promega, USA) and co-transfected into PB or PB-*Sp5* 293T cells with a Renilla luciferase plasmid (Promega, USA). Cells were harvested after 48 h and the luciferase activity of the lysate was measured using the Dual-Luciferase Reporter Assay System (Promega, USA).

### Chromatin immunoprecipitation (ChIP) assay

ChIP assays were performed as previously described [27, 28]. Briefly, PB or PB-*Sp5* mESCs were grown to near confluency in 15-cm dishes. Cells were fixed in 1% formaldehyde. Sheared chromatin was prepared, precleared with protein G-agarose, and immunoprecipitated with anti-HA antibody overnight at 4°C. Immune complexes were captured using protein G-agarose and formaldehyde cross-links in the eluted complexes were reversed. DNA was analyzed by real-time PCR. All related sequences are included in S2 Table (as described in detail previously [29]).

## Results and discussion

### Identification of Sp5 downstream targets in mESCs

**Fig 1:**
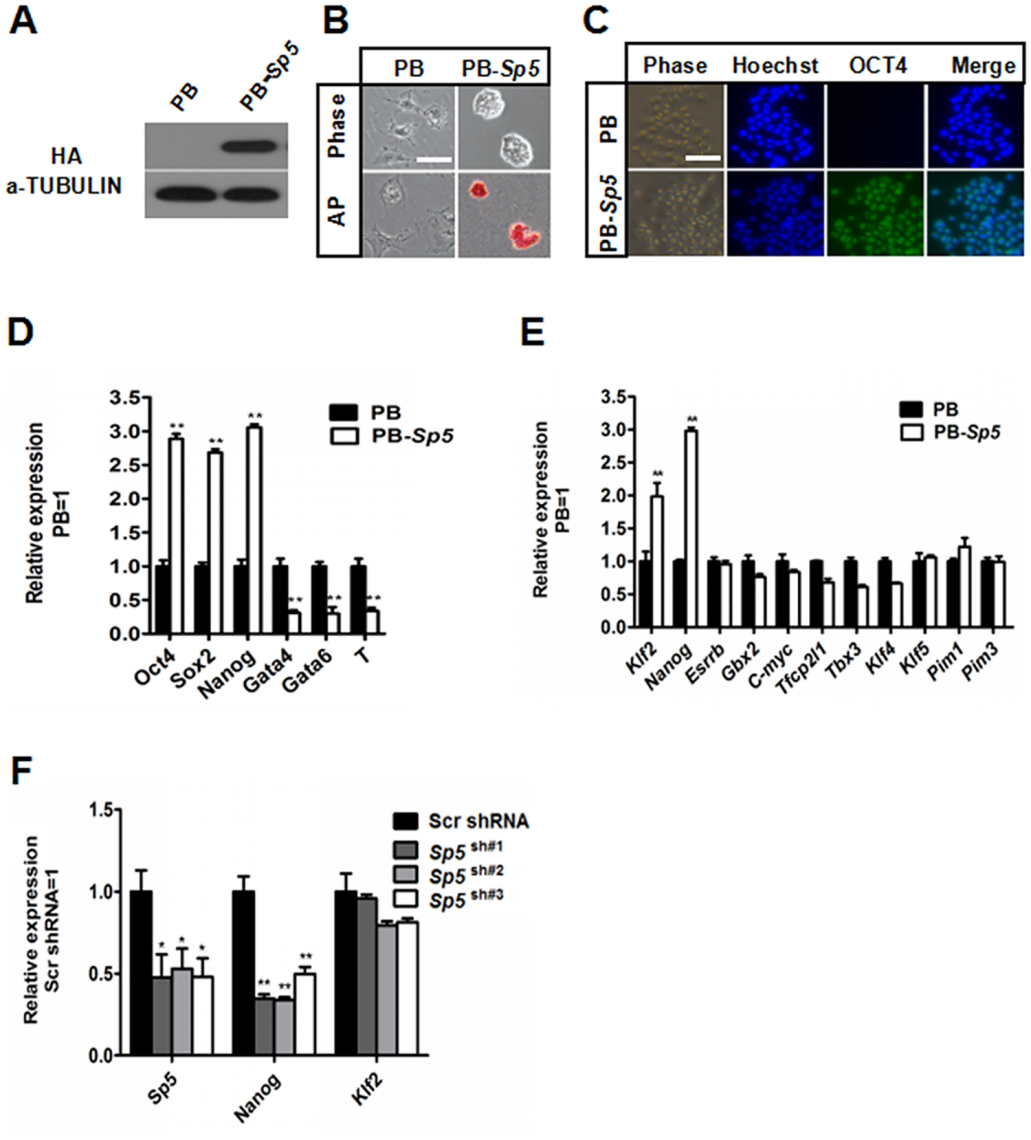
Screening the downstream pluripotency genes regulated by *Sp5*. (A) HA-tagged *Sp5* was introduced into 46C mESCs and the protein level of HA-tagged *Sp5* was determined by Western blot. α-Tubulin was used as a loading control. (B) Phase-contrast and alkaline phosphatase (AP) staining images of PB and PB-*Sp5* mESCs cultured under serum-containing conditions in the absence of LIF for eight days. Scale bar, 100 μm. (C) Immunofluorescence of PB and PB-*Sp5* mESCs cultured under basal media conditions in the absence of LIF. Scale bar, 100 μm. (D) qRT–PCR analysis of the expression levels of mESC pluripotency markers (*Oct4*, *Sox2*, and *Nanog*) and differentiation-associated genes (*Gata4*, *Gata6*, and *T*) in PB and PB-*Sp5* 46C mESCs cultured in the absence of LIF. Data represent the mean±s.d of three biological replicates. **p < 0.01 vs PB. (E) qRT-PCR analysis of *Klf2/4/5*, *Nanog*, *Esrrb*, *Gbx2*, *Myc*, *Tfcp2l1*, *Tbx3* and *Pim1/3* expression in PB and PB-*Sp5* 46C mESCs cultured under LIF/serum-containing conditions. Data represent the mean±s.d. of three biological replicates. **p < 0.01 vs PB. (F) qRT–PCR analysis of *Sp5*, *Nanog* and *Klf2* transcripts in *scramble* and *Sp5* shRNA mESCs cultured under LIF/serum-containing conditions. Data represent the mean±s.d. of three biological replicates. *p < 0.05, **p < 0.01 vs *scramble* shRNA control.

To facilitate the identification of Sp5 targets that contribute to mESC self-renewal, we transduced HA-tagged *Sp5* (PB-*Sp5*) using the PiggyBac (PB) transposon-based vector into 46C mESCs and Western blot analysis was used to confirm the enhanced expression of Sp5 (Fig 1A). After culture in serum-containing medium in the absence of LIF for eight days, PB-*Sp5* mESCs were continuously propagated while retaining typical mESC morphology and alkaline phosphatase (AP)-positive staining (Fig 1B). Immunofluorescence showed positive expression of the pluripotency marker OCT4 (Fig 1C). At the transcriptional level, as assessed by qRT-PCR, overexpression of *Sp5* maintained most of its pluripotency genes (*Oct4*, *Sox2* and *Nanog*), but suppressed the differentiated genes (*Gata4*, *Gata6* and *T*) compared to the PB empty vector (Fig 1D) (as described in detail previously [15]). Many reports have demonstrated that *Klf2/4/5*, *Nanog*, *Esrrb*, *Gbx2*, *c-Myc*, *Tfcp2l1*, *Tbx3* and *Pim1/3* [9, 11, 13, 14, 30–37] can replace LIF to support mESC self-renewal when overexpressed. To investigate whether Sp5 maintains the undifferentiated state of mESCs through regulation of these genes, we used qRT-PCR to detect their expression levels in PB and PB-*Sp5* mESCs cultured under LIF/serum-containing conditions. Overexpression of *Sp5* upregulated *Nanog* and *Klf2* expression, but not that other factors (Fig 1E). To further confirm the two downstream targets of Sp5, we constructed a lentiviral vector expressing *Sp5*-specific shRNA sequences and observed that knockdown of *Sp5* downregulated the *Nanog* transcript, but not *Klf2* (Fig 1F). Additionally, we designed three guide RNAs of *Sp5* to knockout the Sp5 gene in 46C mESCs using the CRISPR/Cas9 system. After selection, we picked and expanded 18 colonies cultured in LIF/serum-containing media. The disruption of both *Sp5* alleles was confirmed in two clones by genomic DNA sequencing (S1 Fig). As expected, the expression of *Nanog* was lower in *Sp5*-null mESCs compared with wild type cells (S1 Fig), but all were maintained in an undifferentiated state and stained positive for AP activity when cultured under LIF/serum-containing conditions (S1 Fig). This is not surprising, as the pluripotency of *Nanog*-null and *Sp5*-knockdown mESCs can be maintained in LIF/serum media [15, 38]. Taken together, these results suggest that Nanog is a downstream target of Sp5.

### Nanog is a direct downstream target of Sp5

**Fig 2:**
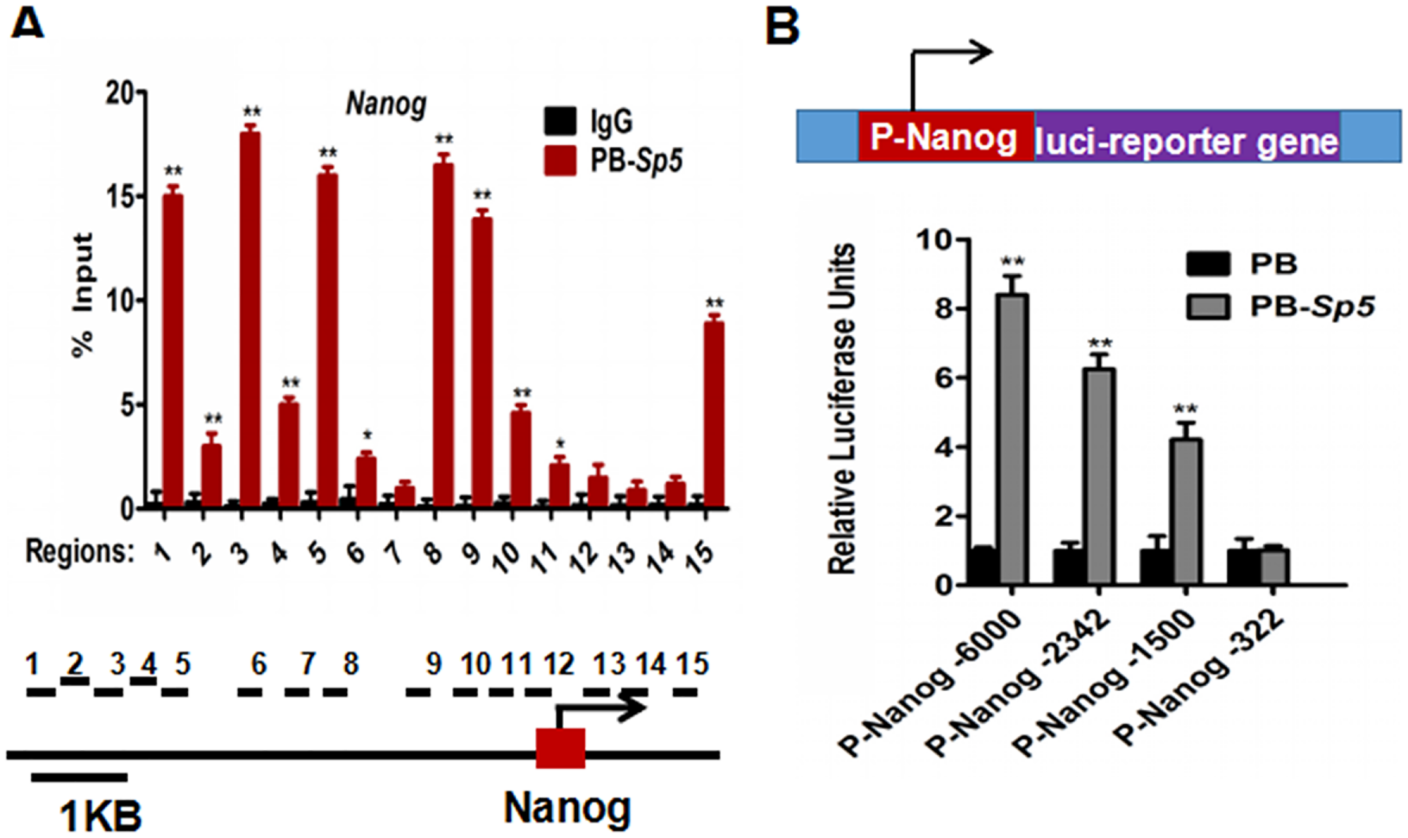
Sp5 directly regulates the transcription of *Nanog*. (A) Independent validation of Nanog as an Sp5-bound target by ChIP-qPCR with fifteen primers set to scan different fragments of the Nanog promoter. Primers set at sites 1, 3, 5, 8, 9 and 15 represent significant enrichment. Data represent the mean±s.d. of three biological replicates. *p < 0.05, **p < 0.01 vs IgG. (B) Schematic illustration of luciferase reporter plasmids and *Sp5* expression activates the P_Nanog_-luciferase reporter. Data represent the mean±s.d. of three biological replicates. **p < 0.01 vs PB.

To further determine whether Nanog is a direct target of Sp5, we performed ChIP-qPCR to examine whether Sp5 directly binds to the Nanog promoter and found that Sp5 was enriched and located in several segments of the Nanog promoter (including sites 1, 3, 5, 8, 9, and 15) (Fig 2A). In support of this, we inserted four different regions of the Nanog promoter, including -6000~0, -2342~0, -1500~0 and -322~0, into the PGL3 vector. These luciferase reporter constructs were co-transfected into 293T cells with PB or PB-*Sp5*, respectively. The luciferase reporter assay showed that the magnitude of Nanog luciferase activity corresponded to the ChIP-qPCR analysis (Fig 2B). Overall, these results indicate that Nanog is a direct target of Sp5 in mESCs.

### Nanog mediates the self-renewal-promoting effect of Sp5

**Fig 3:**
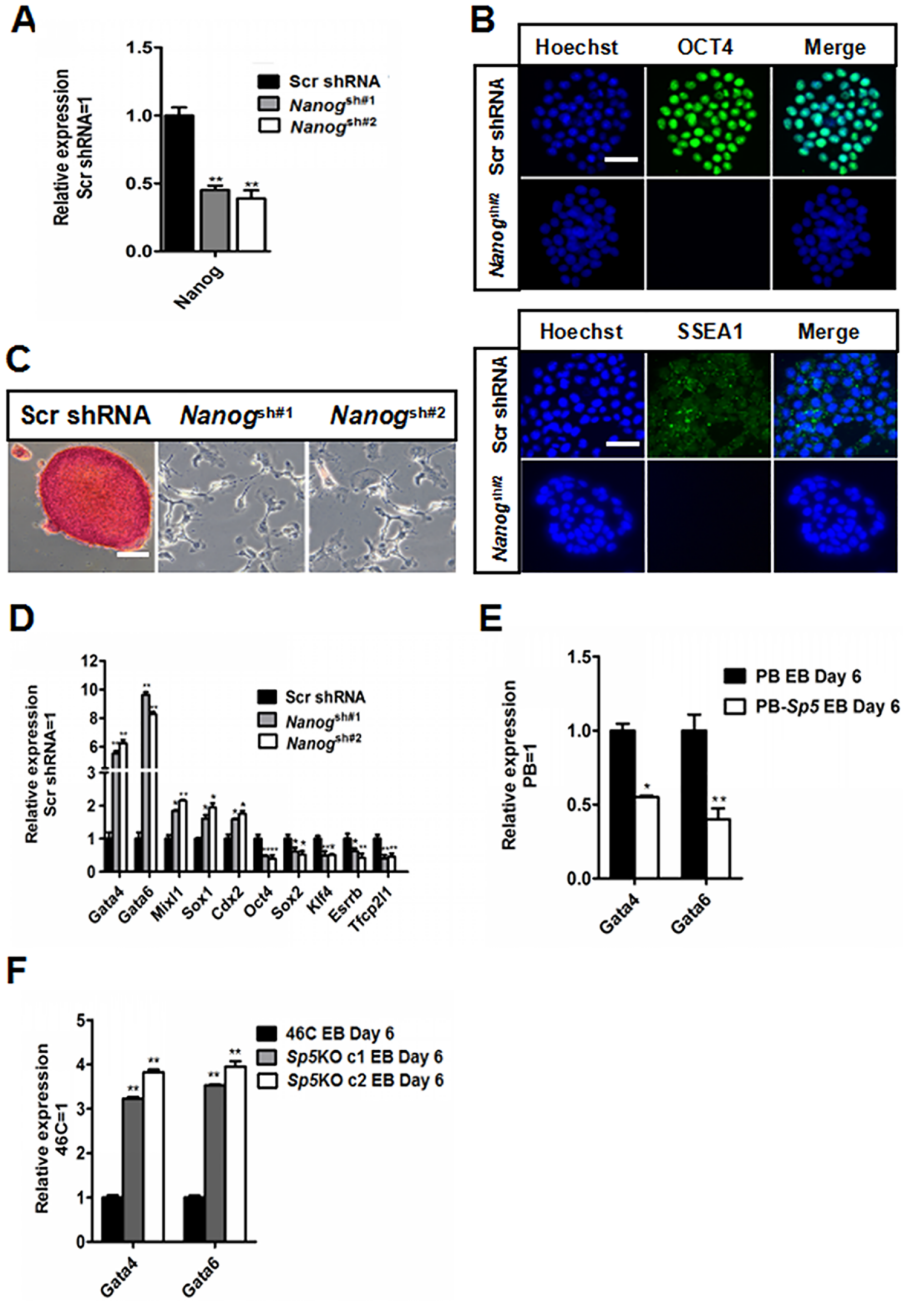
Sp5 relies on Nanog to promote mESC self-renewal. (A) qRT–PCR analysis of *Nanog* expression in PB-*Sp5* mESCs infected with *Nanog* knockdown lentiviruses and cultured in LIF/serum-containing media. The transcript level was normalized to the *scramble* shRNA control. Data represent the mean±s.d of three biological replicates. **p < 0.01 vs *scramble* shRNA control. (B) Immunofluorescence of PB-*Sp5* cells infected with *scramble* control and *Nanog* shRNA lentiviruses cultured under serum-containing conditions in the absence of LIF for eight days. Scale bar, 100 μm. (C) AP staining images of *scramble* control and *Nanog* shRNA mESCs overexpressing PB-*Sp5* cultured under serum-containing conditions. Scale bar, 100 μm. (D) qRT–PCR analysis of the expression of mESC pluripotency markers (*Oct4*, *Sox2*, *Klf4*, *Esrrb* and *Tfcp2l1*) and differentiation-associated genes (*Gata4*, *Gata6*, *Mixl1*, *Sox1* and *Cdx2*) in *scramble* and *Nanog*-knockdown mESCs transfected with PB-*Sp5* cultured in the absence of LIF. Data represent the mean±s.d of three biological replicates. *p < 0.05, **p < 0.01 vs scramble control. (E) qRT–PCR analysis of the expression of *Gata4* and *Gata6* in PB and PB-*Sp5* 46C mESC-derived embryoid bodies harvested on day 6. Data represent the mean±s.d. of three biological replicates. **p < 0.01 vs PB EBs. (F) qRT–PCR analysis of *Gata4* and *Gata6* transcript levels in wild type and S*p5*KO mESC-derived embryoid bodies. Data represent the mean±s.d. of three biological replicates. **p < 0.01 vs 46C EBs.

To investigate whether Nanog mediates the function of Sp5 in promoting mESC self-renewal, we downregulated *Nanog* expression in PB-*Sp5* 46C mESCs with lentiviruses encoding two short-hairpin RNAs (shRNAs) specific to *Nanog* mRNA (Nanog^sh#1^ and Nanog^sh#2^). qRT-PCR analysis confirmed that the *Nanog* transcript level was decreased (50–70%) in these cells (Fig 3A). After culture in serum-containing medium in the absence of LIF for eight days, scramble control-infected PB-*Sp5* mESCs were continually passaged and retained classical mESC morphology, positive AP activity, and expressed the pluripotency markers OCT4 and SSEA1 (Fig 3B and 3C), whereas knockdown of *Nanog* induced differentiation in PB-*Sp5* mESCs, as they expressed lower levels of the pluripotency markers (*Oct4*, *Sox2*, *Klf4*, *Esrrb* and *Tfcp2l1*), but higher levels of the differentiation-associated genes (*Gata4*, *Gata6*, *Mixl1*, *Sox1* and *Cdx2*) compared to scramble control cells (Fig 3D). These results suggest that Sp5 relies on Nanog to promote mESC self-renewal.

Previous reports have shown that *Nanog* inhibits differentiation of the primitive endoderm by repressing the expression of the differentiation markers *Gata4* and *Gata6* in mESCs [33, 34]. To determine whether Sp5 has a similar effect on primitive endoderm specification, we performed an EB formation assay to recapitulate early mouse embryonic development using PB, PB-*Sp5*, wild type 46C or *Sp5* KO mESCs. qRT-PCR analysis showed that PB-*Sp5* efficiently suppressed the expression of primitive endoderm markers (*Gata4* and *Gata6*) compared with PB (Fig 3E). In contrast, *Sp5* KO EBs showed higher levels of *Gata4* and *Gata6* compared to wild type mESC-derived EBs (Fig 3F). These results suggest that Sp5 represses primitive endoderm commitment, similar to Nanog.

Previous reports have also indicated that enhanced *Nanog* can replace LIF to maintain mESC self-renewal [33, 34, 39]. We next constructed a Flag-tagged *Nanog*-overexpressing vector and transfected it into *Sp5* shRNA mESCs (S2 Fig). LIF was then withdrawn from the LIF/serum conditions for eight days to test whether Nanog promotes *Sp5* shRNA mESC self-renewal. These mESCs were continually expanded. Over multiple passages, they exhibited a tightly packed classical mESC morphology and positive AP activity (S2 Fig). qRT–PCR analysis also revealed high-level expression of the pluripotency genes but low-level expression of genes associated with differentiation (S2 Fig). These results further illustrate that Nanog is located downstream of Sp5.

### Sp5 largely depends on its zinc finger domains to maintain mESC self-renewal

**Fig 4:**
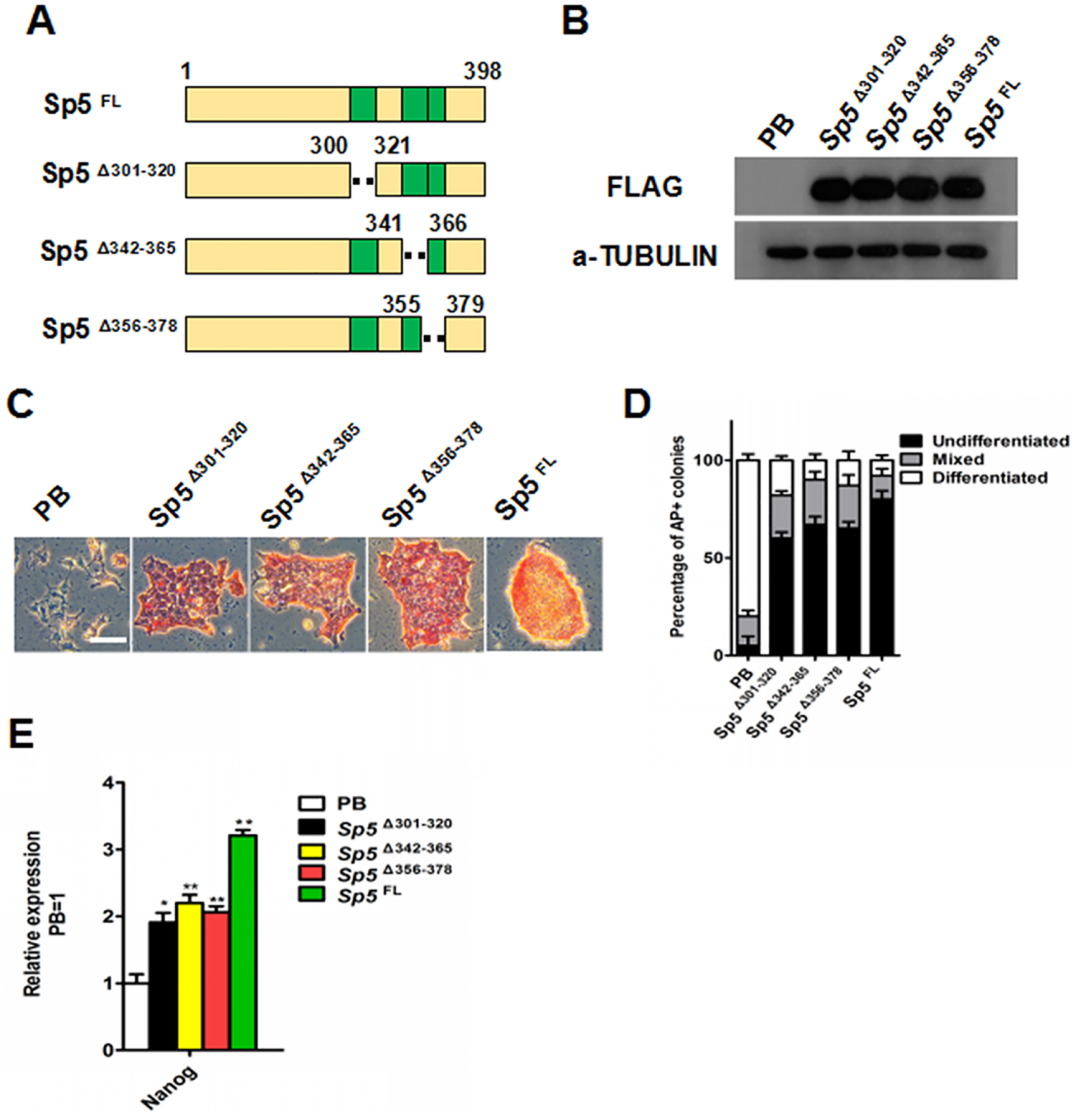
Delineation of the zinc finger domains of Sp5 impairs the self-renewal-promoting effect of Sp5. (A) Schematic illustration of the *Sp5* deletion (Δ) mutants. Zinc finger domains are shown as green boxes. (B) Western blot analysis of Flag-tagged *Sp5* and *Sp5* deletion mutants with anti-FLAG antibody. (C) AP staining images of PB, PB-*Sp5* and *Sp5* deletion mutant mESCs cultured under serum-containing conditions in the absence of LIF for eight days. Scale bar, 100 μm. (D) Quantification of AP-positive colonies in Fig. 4C. (E) qRT–PCR analysis of the expression of *Nanog* in PB, PB-*Sp5* and *Sp5* deletion mutant mESCs cultured in LIF/serum-containing media. Data represent the mean±s.d. of three biological replicates. **p < 0.01 vs PB.

Sp5 belongs to the Sp1 transcription factor family [40, 41] and contains three highly conserved zinc finger domains located near its C-terminus. It is also closely related to the BTEB/KLF gene family [20, 42]. KLF family members, such as *Klf2*, *Klf4* and *Klf5*, have the ability to phenocopy LIF stimulation to maintain the undifferentiated state of mESCs when overexpressed [11, 30–32]. A previous report demonstrated that the C-terminal zinc finger domains are required for the full activity of Klfs and that deletion of two zinc fingers can abolish the efficiency of their self-renewal-promoting activity [43]. However, it remains unknown whether Sp5 has such features. To determine whether the zinc fingers are also required for the self-renewal-promoting effect of Sp5, we generated PB system-mediated expression constructs encoding full-length (FL) and three different mutant mouse Sp5 proteins lacking the first (Δ301–320), second (Δ342–365) or third (Δ356–378) zinc finger (Fig 4A). These Flag-tagged genes were successfully expressed in 46C mESCs, as confirmed by Western blot (Fig 4B). As expected, 46C mESCs transfected with the PB empty vector showed widespread differentiation and no longer expressed AP under serum-containing conditions. However, the full-length and mutant Sp5 transfectants were passaged under serum-containing conditions without overt differentiation for eight days (Fig 4C). Notably, the Sp5 mutant-transfected mESCs produced less AP-positive colonies than the PB-*Sp5*^*FL*^ mESCs and some colonies exhibited a comparatively flatter morphology (Fig 4C and 4D). Finally, to confirm whether the zinc fingers are essential for the full effect of Sp5, we next tested their ability to regulate the expression of *Nanog*. As expected, the deletion mutant exhibited a lower level of *Nanog* expression in *Sp5*-overexpressing mESCs compared to PB-*Sp5*^*FL*^ mESCs (Fig 4E). Taken together, these results suggest that the three zinc finger domains are important for the full activity of Sp5 and that the deletion of each impairs the self-renewal-promoting effect of Sp5 in mESCs.

## Conclusions

Sp5 acts as a target of the LIF/Stat3 signaling pathway and elevated expression of *Sp5* recapitulates the self-renewal-promoting effect of LIF to sustain mESC pluripotency [15]. However, the detailed molecular mechanism by which Sp5 mediates its action on mESC maintenance remains unknown. In this study, we demonstrated that Nanog is the major downstream effector of Sp5 and exerts a unique function downstream of Sp5 that is necessary for the full ability of Sp5 in maintaining mESC self-renewal. In support of this, we presented evidence that knockdown of *Nanog* induces differentiation in *Sp5*-overexpressing mESCs. We also showed that Sp5 depends on its three zinc finger domains to maintain mESC identity. Thus, the intact domains of Sp5 are essential for the Sp5-mediated induction of *Nanog* and maintenance of mESC pluripotency. In the future, understanding how Sp5 interacts with other genes or signaling pathways to induce *Nanog* and how the latter cooperates with other downstream targets of Sp5 to maintain the pluripotent state of mESCs may facilitate the development of new and better culture conditions for the derivation of authentic mESCs from various species.

### Statistical analysis

All data are reported as the mean± s.d. Student’s t-test was used to determine the significance of differences. p<0.05 was considered statistically significant.

## Supporting information

S1 Fig Knockout of *Sp5* does not impair mESC self-renewal. (A) Disruption of *Sp5* by the CRISPR/Cas9 system was verified by sequencing genomic DNA. False regions are shown in red typeface. (B) qRT-PCR analysis of *Nanog* expression in *Sp5*-knockout (KO) 46C mESCs cultured under LIF/serum-containing conditions. The transcript level was normalized to the 46C control. Data represent the mean±s.d. of three biological replicates. **p < 0.01 vs 46C. (C) AP staining images of *Sp5*-KO and 46C control mESCs cultured under serum/LIF-containing conditions for more than five passages. Scale bar, 100 μm. (D) Quantification of AP-positive colonies in S1C Fig.
(TIF)

S2 Fig Overexpression of *Nanog* maintains *Sp5*-knockdown mESC self-renewal in the absence of LIF. (A) Flag-tagged *Nanog* was introduced into 46C mESCs infected with the *Sp5* knockdown (KD) lentivirus. The protein level of Flag-tagged Nanog was determined by Western blot. α-Tubulin was used as a loading control. (B) Phase-contrast and AP staining images of *Sp5*-KD mESCs transfected with PB or PB-*Nanog* cultured under serum-containing conditions in the absence of LIF for eight days. Scale bar, 100 μm. (C) qRT-PCR analysis of the expression of self-renewal genes and differentiation markers in PB and PB-*Nanog* mESCs infected with the *Sp5* KD lentivirus cultured under serum-containing conditions in the absence of LIF. Data represent the mean±s.d. of three biological replicates. *p < 0.05, **p < 0.01 vs PB.
(TIF)

S1 Table List of primers used for qRT-PCR analysis. (XLSX)

S2 Table Primer sequences and locations related to the Nanog promoter region. (XLSX)
