# Supplementary figures and images for: Sp5 induces the expression of Nanog to maintain mouse embryonic stem cell self-renewal
Source: PLoS One. 2017 Sep 29;12(9):e0185714. doi: 10.1371/journal.pone.0185714 (PMC5621696; doi:10.1371/journal.pone.0185714)

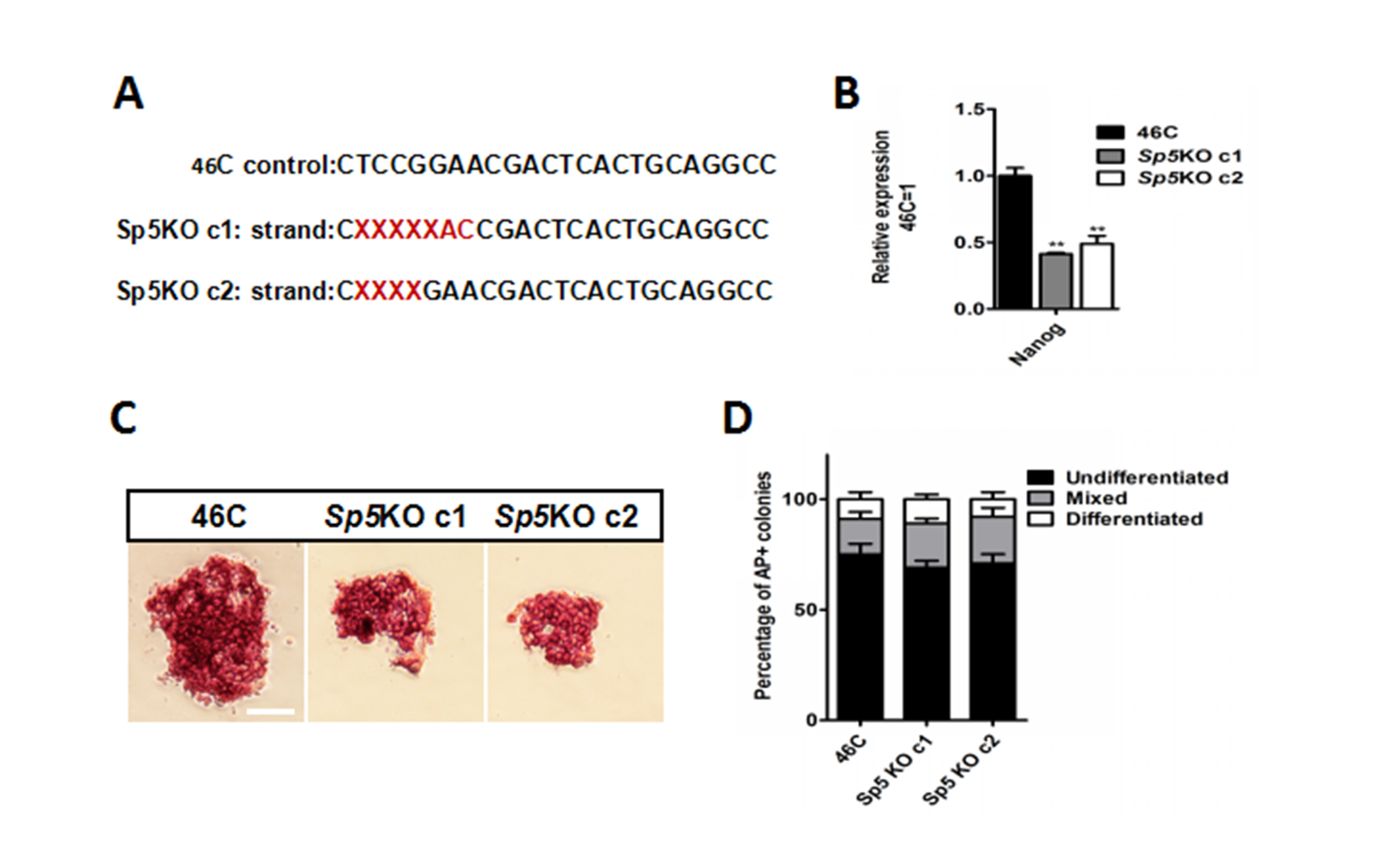

Supplement: S1 Fig — (A) Disruption of Sp5 by the CRISPR/Cas9 system was verified by sequencing genomic DNA. False regions are shown in red typeface. (B) qRT-PCR analysis of Nanog expression in Sp5-knockout (KO) 46C mESCs cultured under LIF/serum-containing conditions. The transcript level was normalized to the 46C control. Data represent the mean±s.d. of three biological replicates. **p < 0.01 vs 46C. (C) AP staining images of Sp5-KO and 46C control mESCs cultured under serum/LIF-containing conditions for more than five passages. Scale bar, 100 μm. (D) Quantification of AP-positive colonies in S1C Fig. (TIF) [file pone.0185714.s001.tif]

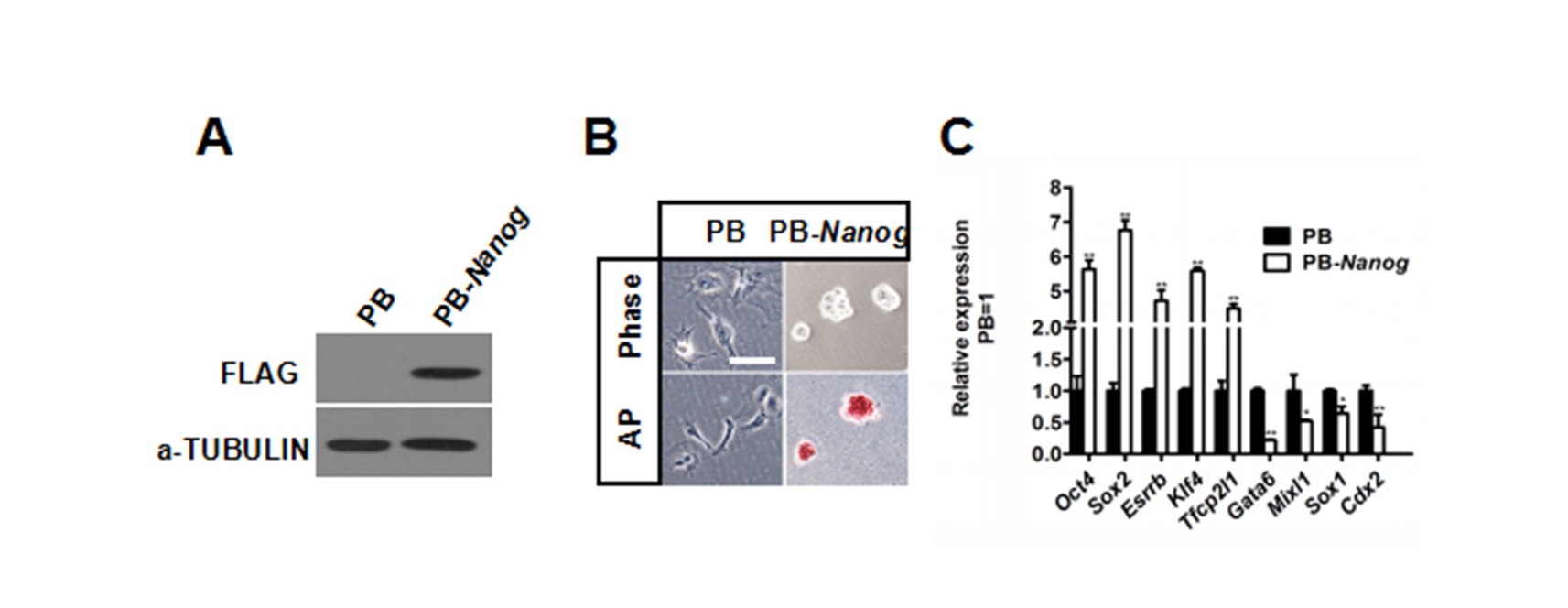

Supplement: S2 Fig — (A) Flag-tagged Nanog was introduced into 46C mESCs infected with the Sp5 knockdown (KD) lentivirus. The protein level of Flag-tagged Nanog was determined by Western blot. α-Tubulin was used as a loading control. (B) Phase-contrast and AP staining images of Sp5-KD mESCs transfected with PB or PB-Nanog cultured under serum-containing conditions in the absence of LIF for eight days. Scale bar, 100 μm. (C) qRT-PCR analysis of the expression of self-renewal genes and differentiation markers in PB and PB-Nanog mESCs infected with the Sp5 KD lentivirus cultured under serum-containing conditions in the absence of LIF. Data represent the mean±s.d. of three biological replicates. *p < 0.05, **p < 0.01 vs PB. (TIF) [file pone.0185714.s002.tif]
